# Compressibility of lime-treated dredged slurry with high water content

**DOI:** 10.1038/s41598-024-63777-3

**Published:** 2024-06-08

**Authors:** Zhen-qi Weng, Xin-yu Huang, Jian-da Chen, Sheng-liang Lu, Ding-yu Ni, Jie-Fei Wang

**Affiliations:** 1 Institute of Geotechnical Engineering, Zhejiang University of Technology, Hangzhou, 310014 China; 2 Wenzhou Polytechnic, Wenzhou, 325035 China; 3 Hangzhou Municipal Construction and Development Group Co., Ltd, Hangzhou, 310014 China

**Keywords:** Lime-treated, Intrinsic compression, Yield stress, Laboratory tests, Civil engineering, Engineering

## Abstract

Lime is widely used for soft ground treatment, rendering the compressibility of lime-treated soil a crucial factor in deformation analysis in engineering applications. This study investigated the compressibility of three remoulded lime-treated slurries with high water content in Southeast China. Sixty groups of oedometer tests were conducted on lime-treated soils with an initial water content of 1 to 3 times the liquid limit and lime contents between 1 and 3%. The oedometer test results were discussed to examine the remoulded yield stress ${\upsigma }_{y}^{\prime}$ of lime-treated slurry. Considering the relationships between ${\upsigma }_{y}^{\prime}$, the void ratio, lime content, and initial water content were preliminarily discussed and quantitatively established. Research on the normalised compression curve of lime-treated soil revealed that for soil samples containing a lime content of 0–%, the normalised compression curve at ${\upsigma }_{p}^{\prime}$>${\upsigma }_{y}^{\prime}$ can be represented by a unique line. Furthermore, the log(1 + *e*) − log ${\sigma }_{\text{v}}^{\prime}$ compression curve of lime-treated slurry at pre-yield state is analysed, and a prediction method for the modified compression index is proposed.

## Introduction

Land reclamation has become the primary countermeasure for addressing land shortages in China’s coastal cities. In the majority of land reclamation projects, dredged sludge from nearby seabeds or river channels is utilised as fill material^1,2^. However, using dredged sludge directly as a building foundation is difficult because of its high water content, low bearing capacity, and high compressibility. Since the early 1960s, lime treatment has been used for the short-term modification of soil properties and has been extensively used in civil engineering applications to stabilise soft clay. It offers numerous benefits, such as easy construction, a short construction period, and cost-effectiveness. Lime treatment is acknowledged as one of the most valuable techniques for improving dredged sludge.

The compressibility of the lime-treated soil is an important issue in deformation analysis for engineering applications. Multiple studies on lime-treated soils have shown that lime treatment effectively reduces compressibility while improving the soil’s bearing capacity^3–10^. For soils with a high water content, lime treatment fosters the formation of a stable soil structure and imparts remoulded yield stress to clay soils^11^. This soil structure curtails the deformation of lime-treated clays under effective vertical stress up to the remoulded yield stress^12–14^.

Several studies have conducted systematic analyses on the changes in compressibility characteristics of soil after lime treatment. Mavroulidou et al.^15^ investigated the impact of lime treatment on the compressibility of high-plasticity clay based on oedometer tests. Sun et al.^16^ studied the compressibility and strength characteristics of lime-treated expansive soil and red clay. Wang et al.^17^ conducted oedometer tests to investigate the compressibility of lime-treated marine soils with 90.9% water content, and found that the difference in compressibility between structured and remolded soils was caused by soil structure and water content. Wang et al.^18^ conducted lime treatment on soil samples with different particle size distributions to study the effect of aggregate size on the compressibility of lime-treated soils. The results show that lime-treated soil samples with a higher content of fine aggregates exhibit greater compressibility. Existing studies have primarily concentrated on the curing mechanisms and the effects of parameters such as lime content and soil properties on the compressibility of lime-treated soil. As far as our knowledge extends, it remains challenging to predict the improvement in compressibility of lime-treated soil, particularly under conditions of high water content. The application of lime treatment in soft ground engineering will be significantly facilitated if a reliable method is established to predict the compressibility of lime-treated dredged slurries.

This paper summarises the research findings on lime treatment for improving the compressibility of high water content remoulded soils from Southeast China. Three soil types were subjected to oedometer tests, varying under different initial conditions of water content (ranging from 1.0 to 3.0 times the liquid limit) and lime content (ranging from 0 to 3% of the dry soil’s weight). The remoulded yield stress of the lime-treated soil was systematically examined using 60 tests, with an analysis of the effects of the initial water and lime contents on the yield stress. The typical behaviour in terms of compressibility is outlined, and an empirical formula for predicting the yield stress of lime-treated soil is developed based on the intrinsic soil properties. Furthermore, the modified compression index *C*_cr_ of the lime-treated slurry was analysed to predict the compressibility in the pre-yield state.

## Materials and methods

### Soil

The study used three distinct types of soil samples, as follows: (1) Wenzhou slurry was obtained from a sea reclamation region in Dongtou County, Wenzhou. The geological context is as follows: The soil layer ranging from − 1.34 to 0.83 m primarily consists of dredged slurry with an initial water content exceeding 100%. (2) Taizhou slurry, acquired from a reclamation site formed through the deposition of soils dredged from Luqiao district, Taizhou. The soil sample originates from the dredged slurry layer (− 6.47 m to 2.41 m). (3) Hangzhou slurry, extracted from a foundation pit at a construction site in Shangcheng District, Hangzhou. The soil sample was obtained from the silt layer (− 1.99 m to − 0.85 m).

**Figure 1:**
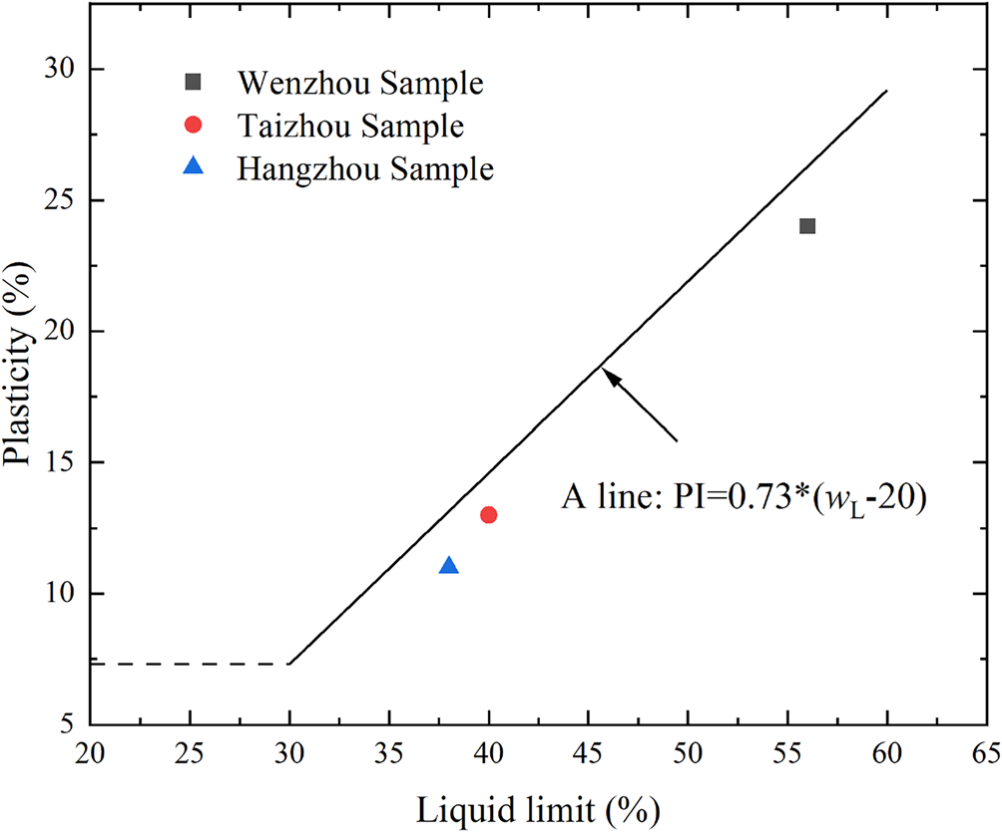
Plasticity chart of the investigated slurries.

**Figure 2:**
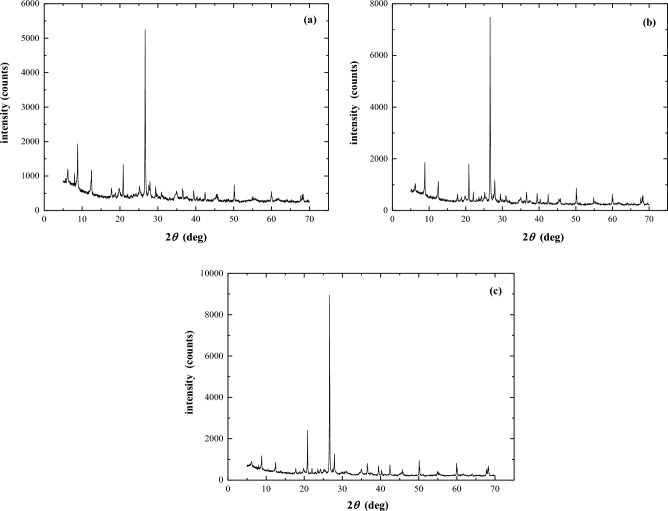
X-ray diffraction patterns of three soil samples. (**a**) Wenzhou slurry, (**b**) Taizhou slurry, (**c**) Hangzhou slurry.

Table 1 summarises the physical properties of the three slurries. The *w*_L_ (liquid limit) of Hangzhou and Taizhou slurry is similar, whereas that of Wenzhou slurry is higher. According to Casagrande’s plasticity chart (Fig. 1), the data point of Wenzhou slurry is located below the A-line defined by PI = 0.73 × (*w*_L_ − 20). This indicates that Wenzhou slurry belongs to the high plasticity silt group. Similarly, the Taizhou and Hangzhou slurries can be classified as silt with low plasticity, using the Unified Soil Classification System (USCS). Figure 2 and Table 2 display the XRD (X-Ray Diffraction) and XRF (X-Ray Fluorescence) experimental results for three types of slurry samples. The experimental results indicate that the mineral composition of the three slurry samples is primarily dominated by primary minerals, with a relatively uniform mineral composition. Quartz has the highest content, followed by kaolinite and illite. The Wenzhou slurry also contains a certain amount of mica, while the Taizhou slurry includes minor amounts of calcite and albite.

**Table 1:** Basic physical properties of the three slurries.

Sample site	Specific gravity	Liquid limit: %	Plastic limit: %	Plasticity index	USCS classification
Wenzhou	2.55	56	32	24	MH
Taizhou	2.63	40	27	13	ML
Hangzhou	2.62	38	27	11	ML

USCS refers to a unified soil classification system; ML refers to silt with low plasticity; MH refers to silt with high plasticity.

**Table 2:** Chemical composition of three slurries.

Chemical composition (% by mass)	Sample site		
	Wenzhou	Taizhou	Hangzhou
SiO_2_	62.39	59.31	66.79
Al_2_O_3_	18.73	20.38	18.26
Fe_2_O_3_	5.44	6.63	4.73
MgO	3.95	4.29	3.41
CaO	3.02	2.74	1.44
K_2_O	2.88	3.19	2.69
Na_2_O	1.89	1.65	1.44
TiO_2_	0.74	0.77	0.74
P_2_O_5_	0.18	0.18	0.15
Loss on ignition	0.78	0.86	0.35

### Hydrated lime

Laboratory-grade hydrated lime, Ca(OH)_2_ (specific gravity 2.21), supplied by Sinopharm Chemical Reagent Co., Ltd., was used in this study. Quantitative analysis by thermal analysis indicated that the lime contained 96% Ca (OH)_2_ and 4% calcite.

### Experimental apparatus

**Figure 3:**
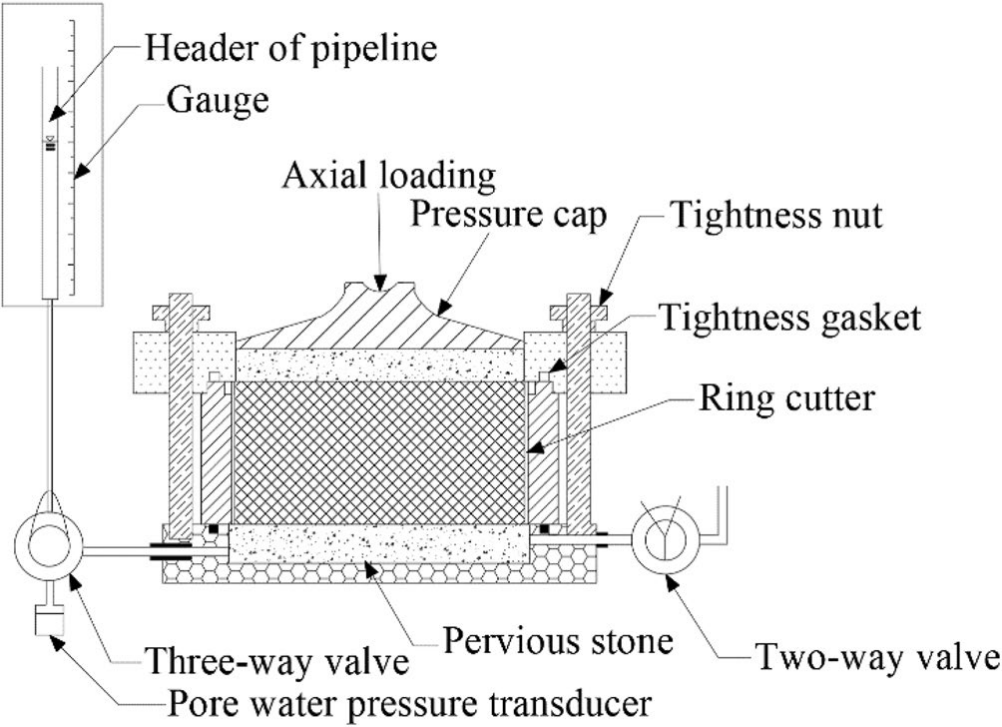
Schematic diagram of one-dimensional osmotic consolidation instrument.

The initial effective vertical stress in the standard consolidation tests is 12.5 kPa in accordance with the standard for the soil test method (ASTM D2435-2011). However, the dredged slurry with a high water content used in this test (water content up to three times the liquid limit) was not sufficiently rigid to endure such a large vertical stress. Therefore, a modified oedometer apparatus with an initial effective vertical stress of 1 kPa was used to conduct the oedometer test of the high water content lime-treated soil. This modified oedometer apparatus was equipped with a light loading cap and two weight hanger systems, as shown in Fig. 3. Information on the device is detailed in Hong and colleagues^15^. The soil samples had a diameter of 6.18 cm and a height of 2 cm. When the effective vertical pressure ranged from 0 to 12.5 kPa, the sample was loaded using the first set of loading systems, positioned at the center point of the consolidation cell. However, when the vertical pressure exceeded 12.5 kPa, the sample was loaded using the second set of loading systems, which could provide higher vertical pressures. The samples were loaded in the following sequence: 1 kPa, 2 kPa, 3 kPa, 4 kPa, 6 kPa, 8 kPa, 10 kPa, 12.5 kPa, 25 kPa, 50 kPa, 100 kPa, 200 kPa, 300 kPa, 400 kPa, and 800 kPa. Each loading process lasted for approximately 24 h.

### Sample preparation

The soil sample used in this test was first air-dried and then passed through a target sieve (D_max_ = 0.4 mm) to remove large impurity particles. All the samples were prepared using the same procedure. A dosage of hydrated lime (designated as 0%, 1%, 2%, and 3% by dry weight of soil) was applied. The soil powder was thoroughly mixed with lime and humidified using distilled water. The initial water content of specimens was adjusted within the range of 1.0–3.0 times their liquid limits (designated as 1.0, 1.5, 2.0, 2.5, and 3.0 times their liquid limits, respectively). Table 3 presents the variable sets for the total of sixty tests. The homogeneous mixture was subsequently transferred to the oedometer rings. After placement in the rings, the soil samples with *w*_0_ = 1.0–1.5 *w*_L_ were soaked under water and then subjected to vacuum for 24 h to facilitate saturation. This vacuum time is typically considered adequate to eliminate the effect of suction on compression. Soil samples exhibiting high water content in the fluid state were considered to be saturated.

**Table 3.** Initial water content and lime content of three slurries.

Soils	Initial water content, *w*_0_: %	Liquid limit, *w*_L_: %	*w*_0_ / *w*_L_	Lime content: %
Taizhou slurry	40	40	1.00	0%,1%,2%,3%
	60	40	1.50	0%,1%,2%,3%
	80	40	2.00	0%,1%,2%,3%
	100	40	2.50	0%,1%,2%,3%
	120	40	3.00	0%,1%,2%,3%
Hangzhou slurry	38	38	1.00	0%,1%,2%,3%
	57	38	1.50	0%,1%,2%,3%
	76	38	2.00	0%,1%,2%,3%
	95	38	2.50	0%,1%,2%,3%
	114	38	3.00	0%,1%,2%,3%
Wenzhou slurry	56	56	1.00	0%,1%,2%,3%
	84	56	1.50	0%,1%,2%,3%
	112	56	2.00	0%,1%,2%,3%
	140	56	2.50	0%,1%,2%,3%
	168	56	3.00	0%,1%,2%,3%

Subsequently, the sample was cured at 22 ℃ for 28 d following saturation. It should be noted that the soil sample remained immersed in water during the curing process.

## Results and discussion

### Compression curve

The selection of the compression relationship had a significant influence on the analysis of soil compression characteristics. The e-log ${\sigma }_{\text{v}}^{{{\prime}}}$ compression relationship is the most frequently used approach for normally consolidated soils. The compression curve of normally consolidated soil is often approximated as either a straight line or a curve with only one knee point in e-log ${\sigma }_{\text{v}}^{{{\prime}}}$ coordinates. For this type of soil sample, the Casagrande method can be used to derive the preconsolidation pressure or yield stress.

**Figure 4:**
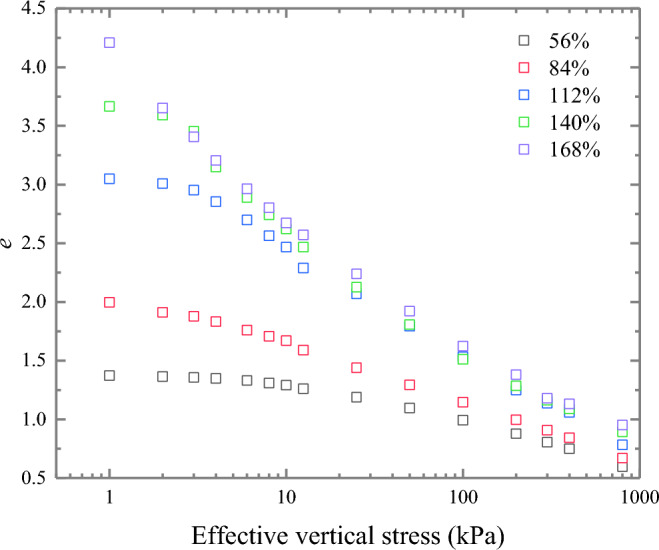
Compression curves of Wenzhou remolded soils (0% lime content) at different initial Water content.

Figure 4 reflects the e-log ${\sigma }_{\text{v}}^{{{\prime}}}$ curves of Wenzhou natural soil samples under various water content conditions. It is evident that for soft clays with high water content, a distinct straight line is not discernible towards the end of the e-log ${\sigma }_{\text{v}}^{{{\prime}}}$ curve. Instead, the overall compression curve assumes an inverted "S" shape, particularly when the water content lies between 100 and 250%^19^. Therefore, the Casagrande method cannot be used to determine the yield stress of soils characterised by high water content.

**Figure 5:**
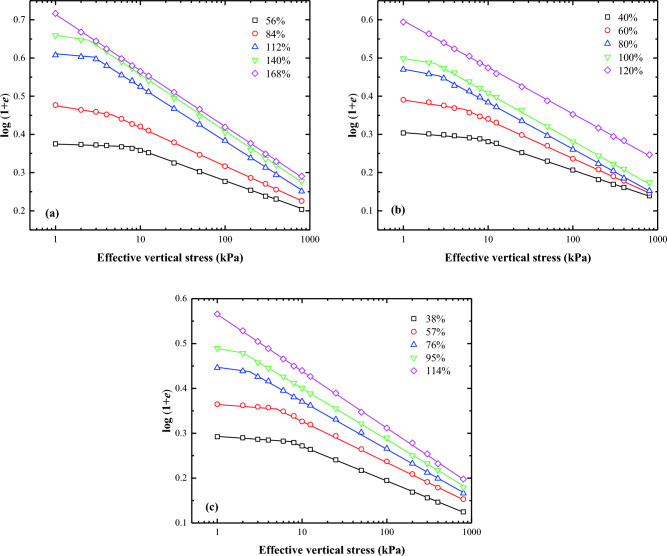
Bilogarithmic compression curves of soils at different initial Water content: (**a**) Wenzhou slurry; (**b**) Taizhou slurry; (**c**) Hangzhou slurry.

**Figure 6:**
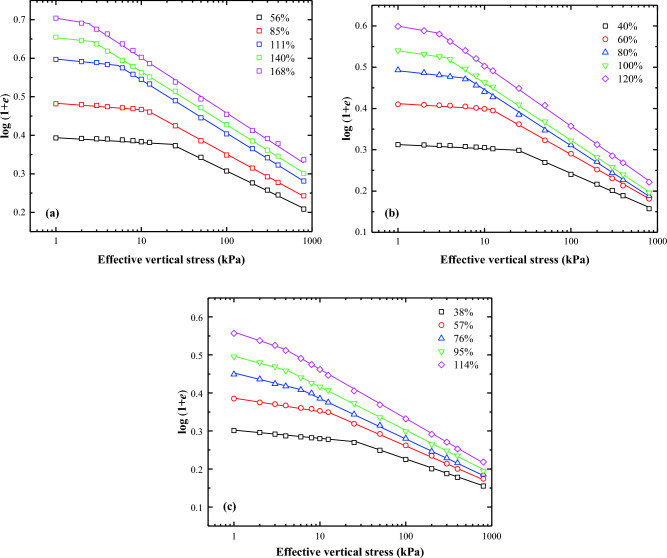
Bilogarithmic compression curves of soils (1% lime content) at different initial Water content: (**a**) Wenzhou slurry; (**b**) Taizhou slurry; (**c**) Hangzhou slurry.

**Figure 7:**
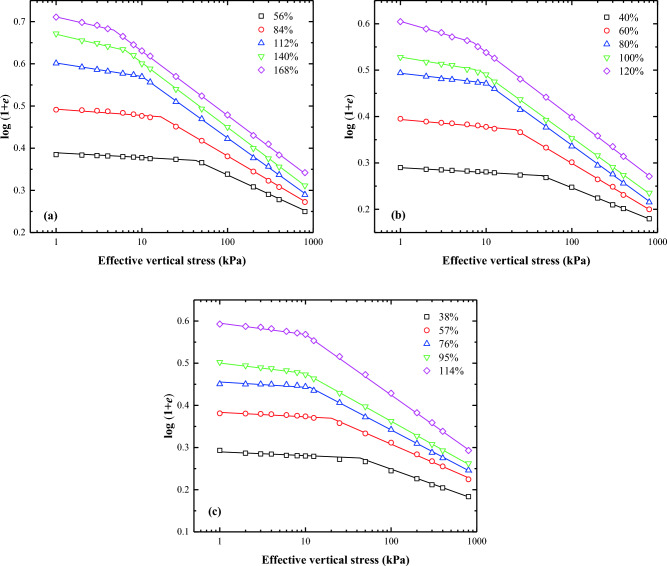
Bilogarithmic compression curves of soils (2% lime content) at different initial Water content: (**a**) Wenzhou slurry; (**b**) Taizhou slurry; (**c**) Hangzhou slurry.

**Figure 8:**
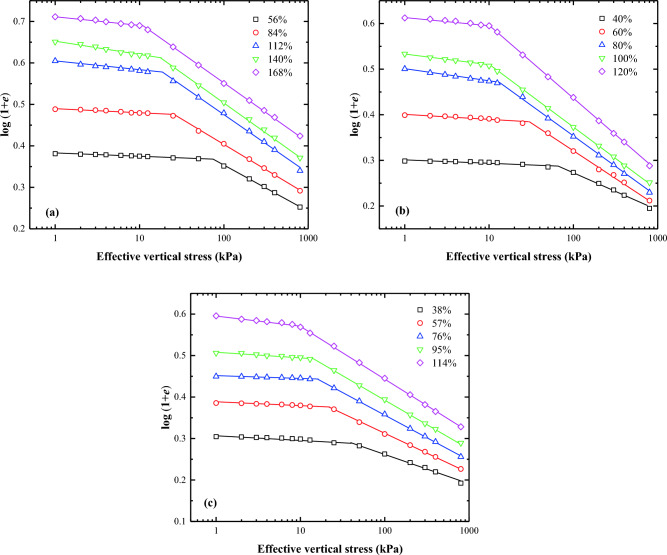
Bilogarithmic compression curves of soils (3% lime content) at different initial Water content: (**a**) Wenzhou slurry; (**b**) Taizhou slurry; (**c**) Hangzhou slurry.

Butterfield^12^ first introduced the bilogarithmic method for interpreting oedometer test data for natural soils. The compression curves of lime treated soil can be effectively represented by two straight lines on a plot of log (1 + *e*) against log ${\sigma }_{\text{v}}^{\prime}$. The effective vertical stress ${\sigma }_{\text{v}}^{\prime}$ at the intersection point of these two straight lines is the remoulded yield stress (${\sigma }_{\text{y}}^{\prime}$). Several researchers, including Onitsuka and colleagues^20^, Sridharan and Prakash^21^, Hong and Onitsuka^22^, and Hong^23^, have validated the accuracy of the proposed method. They demonstrated that the oedometer test data of reconstituted clays could also be plotted as a straight line in the bilogarithmic graph, if the applied vertical stress exceeds ${\sigma }_{\text{y}}^{{{\prime}}}$. Consequently, similar to the interpretation approach for natural soils, the oedometer test data for the reconstituted clays can be represented by two straight lines in the bilogarithmic graph. Figures 5, 6, 7, and 8 exhibit the logarithmic compression curves of the three remoulded slurries with varying lime content. It is observed that most of the oedometer test data can be adequately represented by two straight lines in the log (1 + *e*) − log ${\sigma }_{\text{v}}^{\prime}$ coordinate, except for soil samples with a 0% lime content and a water content of three times the liquid limit. These exception cases can be approximated by a straight line. The stress values at the intersection of the two straight lines correspond to the yield stress. The left straight line corresponds to the pre-consolidation state when ${\sigma }_{\text{v}}^{\prime}$ < ${\sigma }_{\text{y}}^{\prime}$, whereas the right straight line correspond to the post-consolidation state line when ${\sigma }_{\text{v}}^{\prime}$ > ${\sigma }_{\text{y}}^{\prime}$.

In the pre-yield state (${\sigma }_{\text{v}}^{\prime}$ < ${\sigma }_{\text{y}}^{\prime}$) where the vertical effective stress is less than the yield stress, the compressibility of the sample is relatively low. Once the vertical effective stress surpasses the yield stress, a significant increase in compressibility is observed, to the extent that the compression modulus may exceed that of untreated samples. The compressibility of lime-treated soil increases with the addition of lime content and decreases with an increase in moisture content.

### Remolded yield stress

Table 4 lists the yield stresses of the three soil samples, each with varying lime and water contents. The test results showed that soil’s yield stress increased with an increase in lime content, whereas it decreased with an increase in water content. This phenomenon can be attributed to the ion-exchange reaction between the calcium ions in the calcium hydroxide and the sodium ions on the soil particle surface that occurred after the lime was mixed into the soil sample. Ion exchange reduces the thickness of the double-electron layer on the surface of the soil particles, prompting their agglomeration into floccules. With the long-term action of lime, a pozzolanic reaction between excess lime and soil particles produces calcium silicate hydrate (CSH), and calcium aluminate hydrate (CAH) gels with lower water content, which further enhances the strength of the soil. However, an increase in soil water content reduces the concentration of calcium ions and affects the alkaline environment inside the lime-treated soil, resulting in a decrease in the lime treatment effect.

**Table 4.** Remolded yield stress of three soil sample with different lime content.

Sampling location	Lime content	1.0-time liquid limit	1.5 times liquid limit	2.0 times liquid limit	2.5 times liquid limit	3.0 times liquid limit
Wenzhou	Natural sample	8.24	5.12	2.92	2.63	-
	1% lime content	21.67	10.81	5.37	3.27	2.55
	2% lime content	43.97	16.39	9.94	6.40	4.68
	3% lime content	72.02	23.88	20.06	17.62	10.71
Taizhou	Natural sample	8.00	5.26	2.66	2.26	-
	1% lime content	24.74	11.53	5.83	3.72	3.04
	2% lime content	44.38	22.56	9.90	8.68	6.94
	3% lime content	70.96	30.34	14.61	10.18	9.85
Hangzhou	Natural sample	7.49	5.02	2.53	2.11	-
	1% lime content	32.01	12.24	6.51	4.28	3.25
	2% lime content	39.06	18.92	14.08	11.84	9.93
	3% lime content	40.57	20.34	16.00	14.04	9.97

Existing data also suggest that soil water content may affect the lime modification optimum (LMO) of soil. The reinforcing effect of lime treatment on soil does not indefinitely increase with increasing lime content but begins to decrease slowly beyond a threshold value. This threshold value, called the lime modification optimum, was first introduced by McCallister and Petry^20^ through their leaching tests conducted on lime-treated clay. Using the Hangzhou soil sample as an example, when the lime content increases from 1 to 2%, the yield stress of the samples with varying water contents increased by 22.0%, 54.6%, 116.3%, 176.6%, and 205.5%, respectively. This observation highlights that with increasing water content, the effect of lime treatment on the remodelled yield stress becomes evident.

Furthermore, the influence of lime content on the remoulded yield stress was analysed. When the lime content was increased further (3%), the increase in remoulded yield stress decreased significantly. This indicates that the optimal lime content of the Hangzhou slurry is 2%. However, for the remaining samples, the lime content needs to be further increased to determine the optimal lime content.

### Statistical analysis of remolded yield stress

Hong^23^ showed that the void ratio at the liquid limit (${e}_{L}$) can serve as an index for standardising the relationships between the initial void ratio ${e}_{0}$ and yield stress for various reconstituted clays. According to Hong’s investigation^19^, a unique relationship between yield stress and ${e}_{0}/{e}_{L}$ was established for remoulded clay samples with initial water content in the range of 0.7–2.0 times the liquid limit. The following formula was used to describe this relationship:

1 $${\upsigma }_{y}^{\prime}={\upsigma }_{yL}/{({e}_{0}/{e}_{L})}^{2}$$

When ${\upsigma }_{y}^{\prime}$ is the remoulded yield stress, ${e}_{0}$ is the initial void ratio, ${e}_{L}$ is the void ratio at liquid limit and ${\upsigma }_{yL}$ is the yield stress for various reconstituted soils at their liquid limits.

**Figure 9:**
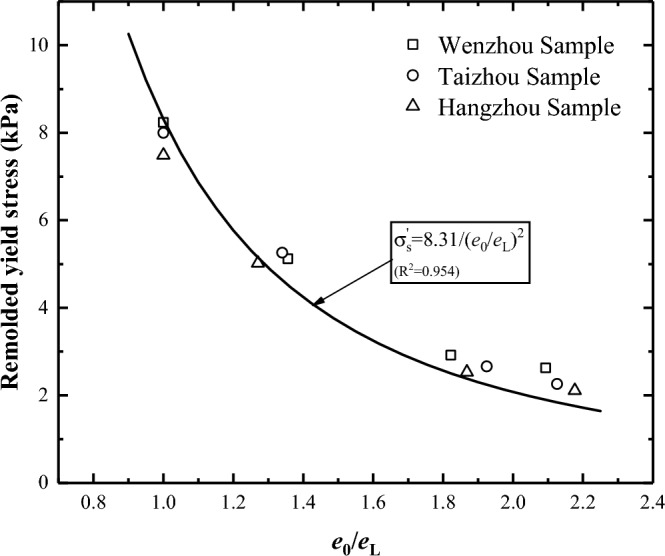
Relationship between remoulded yield stress and normalised initial void ratio.

In this study, this formula was used to analyse the relationship between the yield stresses and pore ratio of the three silts. The results revealed that this formula yielded a good fitting effect on the silts as well. Regression analysis yielded the following simple equation with a correlation coefficient R^2^ of 0.966 (Fig. 9):

2 $${\upsigma }_{y}^{\prime}=8.31/{({e}_{0}/{e}_{L})}^{2}$$

The value of ${\upsigma }_{yL}$ in this formula is 8.31 kPa, which surpasses the value reported by Hong^19^. The rationale for this discrepancy is that the soil sample used in this study was silt and had a substantially higher sand content (particle diameter greater than 0.075 mm) than the soil sample used in Hong^19^. The friction angle of the sample increases as the proportion of sand particles rises, ultimately increasing the pre-consolidation of the soil^24^.

Previous research has shown that lime treatment exerts a significant influence on the plasticity index of soil. The soil’s liquid limit rises with increasing lime content^25^. This can probably be attributed to the substitution of Ca^2+^ with Na^+^ and K^+^ in clay minerals, leading to a reduction in the soil water content^26^. To capture the effect of the lime treatment on the intrinsic properties of a given soil, the parameter $L$ was introduced in the empirical formula, which represents the effect of the lime treatment on the soil’s intrinsic properties such as soil strength, plasticity index, and void ratio at the liquid limit:

3 $${\upsigma }_{y}^{\prime}={\upsigma }_{yL}/{(L \times (\frac{{e}_{0}^{\prime}}{{e}_{L}}))}^{2}$$

where ${\upsigma }_{y}^{\prime}$ is the yield stress of the lime-treated soil, ${e}_{0}^{\prime}$ is the initial void ratio of the lime-treated soil. The parameter ${\upsigma }_{yL}$, set at 8.31, represents the yield stress of the initial soil sample at the liquid-limit void ratio. The fitting formula was as follows:

4 $${\upsigma }_{y}^{\prime}=8.31/{(L \times (\frac{{e}_{0}^{\prime}}{{e}_{L}}))}^{2}$$

**Figure 10:**
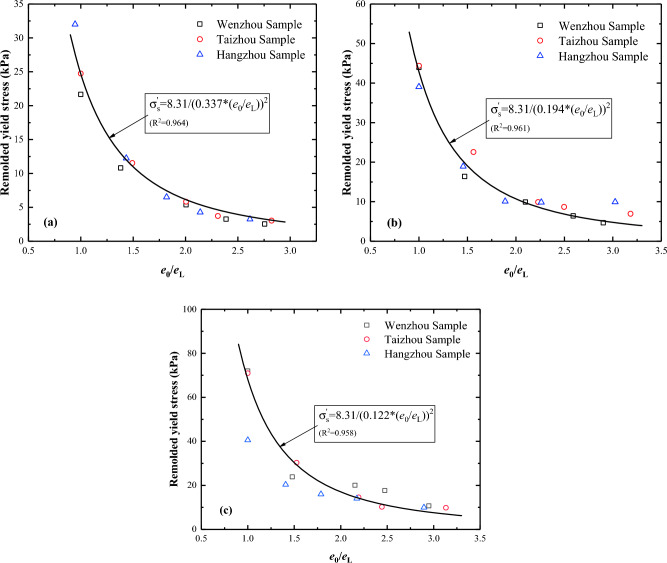
Relationship between yield stress of lime-treated soils and normalised initial void ratio: (**a**) 1% lime content; (**b**) 2% lime content; (**c**) 3% lime content.

The yield stress of the lime-treated soils with different water contents was fitted. The fitting results are shown in Fig. 10. The new formula fits better the yield stress of lime-treated soil, and the correlation coefficients of the fitting formula for the 1%, 2%, and 3% lime contents are 0.963, 0.941, and 0.984, respectively.

### Normalising compression curves of lime-treated slurry

Burland^14^ proposed a normalising index, the void index *I*_v_, to normalise the compression curves of remoulded clay. The expression for the void index *I*_v_ is

5 $${I}_{\text{v}}=(e-{e}_{100}^{*})/({e}_{100}^{*}-{e}_{1000}^{*})=(e-{e}_{100}^{*})/{C}_{\text{c}}^{*}$$

where ${e}_{100}^{*}$ and ${e}_{1000}^{*}$ are the porosity index of the remodelled silt when the vertical effective stress is 100 kPa and 1000 kPa, respectively; ${C}_{\text{c}}^{*}$ is called the inherent compression index, equal to (${e}_{100}^{*}-{e}_{1000}^{*}$).

Based on the void index, Burland^14^ introduced a normalised compression curve termed the intrinsic compression line (ICL). Hong and colleagues^15^ researched the intrinsic compression line of remoulded soil characterised by high water content and proposed an EICL to clarify the compression properties under high water content. EICL refers to the extended intrinsic compression line, which extends the ICL of Burland^14^ to an effective vertical stress as low as 1.5 kPa. Hong and colleagues^15^ suggested that the EICL should be used for the remoulded soil with *w*_L_ = 28–100% and *w*_0_ = 0.7–2.2 *w*_L_.

**Figure 11:**
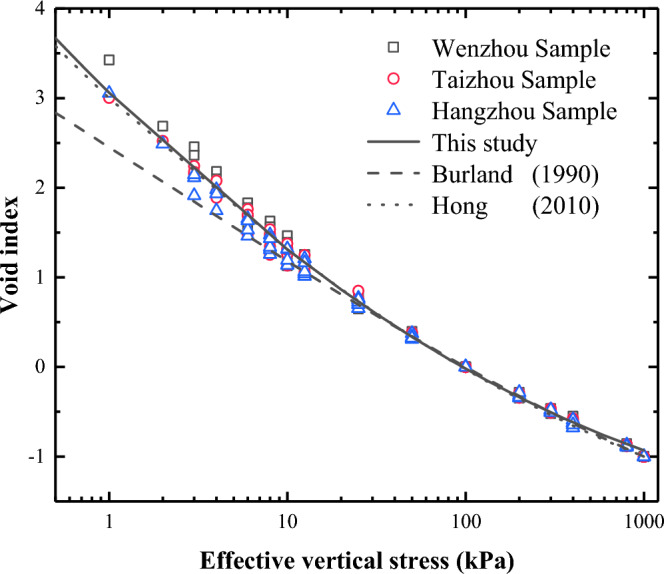
Comparison of normalised compress curves with ICL and EICL.

This paper analysed the EICL of three kinds of remoulded silt with *w*_L_ = 38–56% and *w*_0_ = 1.0–3.0 *w*_L_ over the stress range from the remoulded yield stress to 800 kPa. And the expression is:

6 $${I}_{\text{v}}=3.05-1.959\times \text{log}{\sigma }_{\text{v}}^{\prime}+0.208\times {\left(\text{log}{\sigma }_{\text{v}}^{\prime}\right)}^{2}$$

where ${\sigma }_{\text{v}}^{\prime}$ is vertical effective stress. Figure 11 shows the relationship between the void index and effective vertical stress for the three types of remoulded soil and compares the normalised compression curves in this study with ICL and EICL. The line in this study is almost identical to the EICL proposed by Hong^15^, and is located above the ICL.

**Figure 12:**
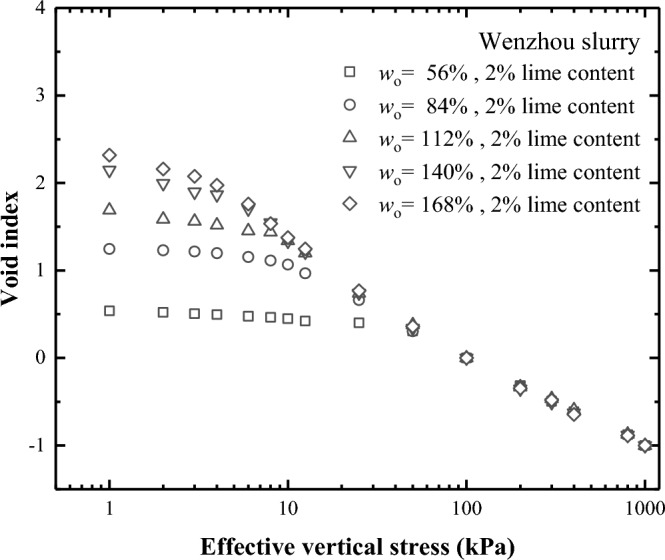
Relationship of void index plotted against effective vertical stress for lime-treated slurry with different initial water content.

**Figure 13:**
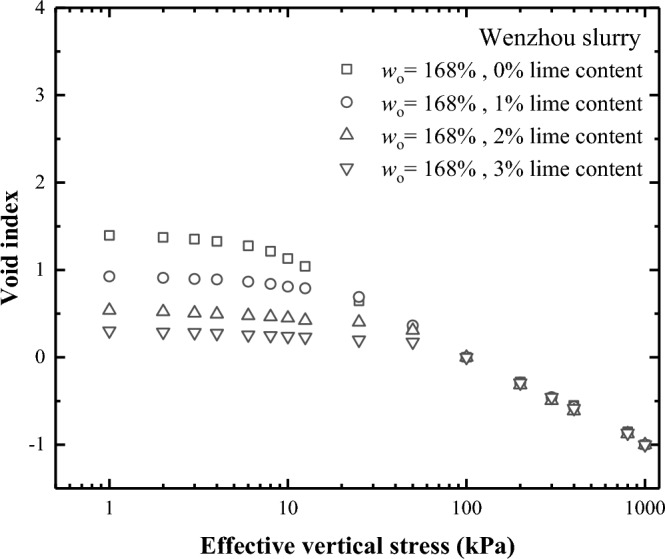
Relationship of void index plotted against effective vertical stress for lime-treated slurry under different lime content.

Figures 12 and 13 depict the correlation between the void index and vertical effective stress for lime-treated slurry across varying initial water and lime contents. The *I*_v_-log ${\sigma }_{\text{v}}^{\prime}$ compression curve of lime-treated soil is similar to that of remoulded soil. The compression curve of the lime-treated slurry with higher initial water content aligns beneath the curve representing lower water content. Beyond a vertical effective of 50 kPa, *I*_v_-log ${\sigma }_{\text{v}}^{\prime}$ compression curves for lime-treated slurry with different water content tend to normalise into a line, which is similar to the findings of Burland^14^ and Hong^19^. With an increase in lime content, the void index of the lime-treated slurry decrease under lower vertical stress conditions. However, when the vertical effective stress surpasses a certain threshold, the *I*_v_-*p* curves tended to be consistent across different lime contents.

**Figure 14:**
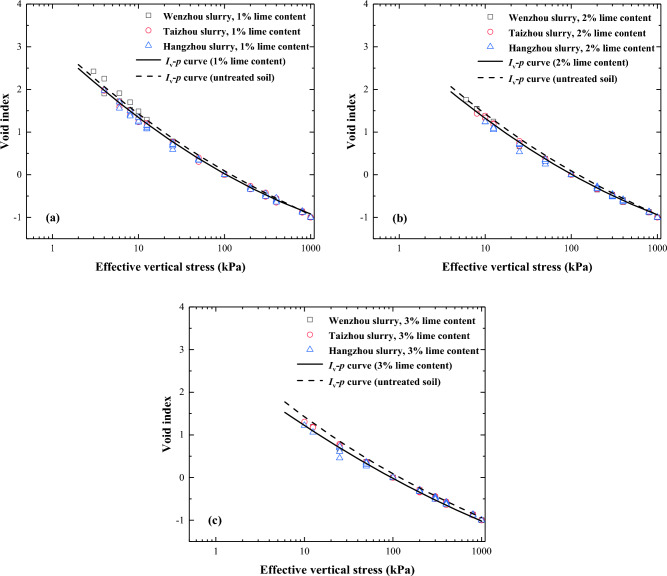
Normalised compression curves for the lime-treated slurry with different lime content (**a**) 1% lime content, (**b**) 2% lime content, (**c**) 3% lime content.

Figure 14 shows the normalised compression curve of the lime-treated slurries with varying lime contents over the stress range from the remoulded yield stress (${\upsigma }_{y}^{\prime}$) to 800 kPa. The normalised compression curves for the lime-treated slurries with 1%, 2%, and 3% lime content are as follows:

7 $${I}_{\text{v}}=3.038-1.888\times \text{log}{\sigma }_{\text{v}}^{\prime}+0.187\times {\left(\text{log}{\sigma }_{\text{v}}^{\prime}\right)}^{2} \left(1\% \; \text{lime content}\right)$$

8 $${I}_{\text{v}}=2.986-1.852\times \text{log}{\sigma }_{\text{v}}^{\prime}+0.178\times {\left(\text{log}{\sigma }_{\text{v}}^{\prime}\right)}^{2} \left(2\%\; \text{lime content}\right)$$

9 $${I}_{\text{v}}=2.702-1.599\times \text{log}{\sigma }_{\text{v}}^{\prime}+0.125\times {\left(\text{log}{\sigma }_{\text{v}}^{\prime}\right)}^{2} \left(3\% \; \text{lime content}\right)$$

The squares of the correlation coefficient (R2) are both above 0.99.

**Figure 15:**
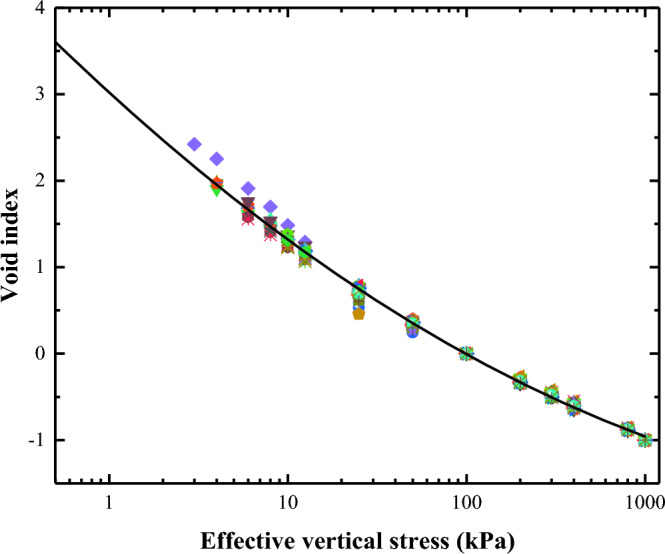
Normalized compression curve of lime-treated slurry.

A detailed analysis of the normalised compression curves of the lime-treated slurries with varying lime contents showed that the three curves were essentially consistent (Fig. 15). Consequently, a unified analysis of the compression curves of slurry samples with under various conditions (soil samples: Wenzhou soil, Hangzhou soil, Taizhou soil; initial moisture content: 56%, 84%, 112%, 140%, 168%; lime content: 1%, 2%, 3%) was performed, and a normalised compression curve of high water content slurry suitable for 1–3% lime content was obtained.

$${I}_{\text{v}}=3.020-1.887\times \text{log}{\sigma }_{\text{v}}^{\prime}+0.187\times {\left(\text{log}{\sigma }_{\text{v}}^{\prime}\right)}^{2}$$

### Compressibility of lime-treated slurry at pre-yield state

The normalised compression curve aptly characterises the compressibility of lime-treated slurry when ${\sigma }_{\text{v}}^{\prime}$ > ${\sigma }_{\text{y}}^{\prime}$. However, it is essential to highlight that the compression curves of lime-treated slurries when ${\sigma }_{\text{v}}^{\prime}$ < ${\sigma }_{\text{y}}^{\prime}$ cannot be normalised into a unique line. Because the remoulded yield stress increased significantly following the lime treatment, the normalised compression curves are not able to portray the compressibility exclusively of the lime-treated slurry. Therefore, it is imperative to analyse the compression curve of lime-treated slurry when ${\sigma }_{\text{v}}^{\prime}$ < ${\sigma }_{\text{y}}^{\prime}$, and propose a simple method to predict the compression curve at the pre-yield state.

In this study, bilogarithmic compression curves were used to analyse the compressibility of lime-treated slurries. The log(1 + *e*) − log ${\sigma }_{\text{v}}^{\prime}$ compression model is expressed as follows:

10 $$\text{log}\left(1+e\right)=\text{log}\left(1+{e}_{0}\right)-{C}_{cr}\text{log}(p/{p}_{0})$$

where ${C}_{cr}$ is the modified compression index, that is, the slope of the straight-line segment of the log(1 + *e*)  − log ${\sigma }_{\text{v}}^{\prime}$ compression model. Figure 3 shows that the log(1 + *e*)  − log ${\sigma }_{\text{v}}^{\prime}$ compression curve of lime-treated soil is composed of two straight lines. This section primarily analyses the straight-line segment when ${\sigma }_{\text{v}}^{\prime}$ < ${\sigma }_{\text{y}}^{\prime}$. The slope of this section is expressed as *C*_*cr*1_.

Table 5 lists the *C*_*cr*1_ values of the Wenzhou slurry for varying lime and initial water contents. The *C*_*cr*1_ of the lime-treated slurry increased with increasing initial water content and decreased with increasing lime content.

**Table 5.** The *C*_*cr*1_ values of Wenzhou slurry after lime treatment.

Initial water content	Lime content			
	0%	1%	2%	3%
*w*_0_ = 1.0 *w*_*L*_	0.0129	0.0106	0.0066	0.0056
*w*_0_ = 1.5 *w*_*L*_	0.0189	0.0154	0.0142	0.0098
*w*_0_ = 2.0 *w*_*L*_	0.0210	0.0207	0.0252	0.0131
*w*_0_ = 2.5 *w*_*L*_	0.0229	0.0349	0.0398	0.0136
*w*_0_ = 3.0 *w*_*L*_	–	0.0436	0.0401	0.0224

**Figure 16:**
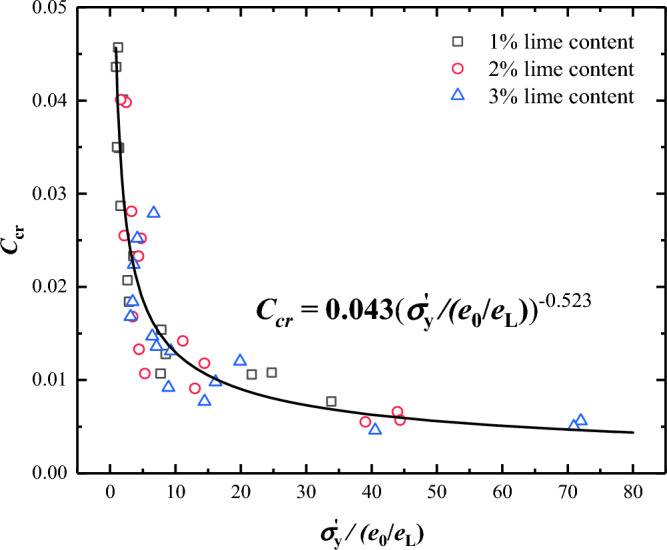
Relationship between ${\sigma }_{y}^{\prime}/({e}_{0}/{e}_{\text{L}})$ and* C*_*cr*1_.

We further analysed the relationship between *C*_*cr*1_ and the soil parameters. It is widely accepted that the value of *C*_*cr*1_ is directly related to the degree of the cementation bond, as reflected by ${\sigma }_{\text{y}}^{\prime}$^27,28^. Normalised parameters ${\sigma }_{y}^{\prime}/({e}_{0}/{e}_{\text{L}})$ were used in this study to analyse the *C*_*cr*1_ of the lime-treated slurry. Figure 16 shows the relationship between ${\sigma }_{y}^{\prime}/({e}_{0}/{e}_{\text{L}})$ and* C*_*cr*1_, which can be expressed as:

11 $${C}_{cr1}=0.043{\left({\sigma }_{y}^{\prime}/({e}_{0}/{e}_{\text{L}})\right)}^{-0.523}$$

## Validation

In order to validate the *I*_v_-*p* curves of lime-treated soil obtained in the present work, additional test data were compared with the formula. The oedometer test data of five soil samples are taken from previous studies^29–32^. The basic properties of the soil samples used in the validation test are shown in Table 6.

**Table 6.** Basic physical properties of test samples.

Sample	Specific gravity	Liquid limit: %	Plastic limit: %	Plasticity index
Lanzhou sample	2.73	28.3	16.5	11.8
Oufei sample	2.61	81	42	39
Karnataka sample	2.67	72.1	31.7	40.4
Al-Ghat sample	2.85	66	32	34
Al-Qatif sample	2.77	158	54	104

**Figure 17:**
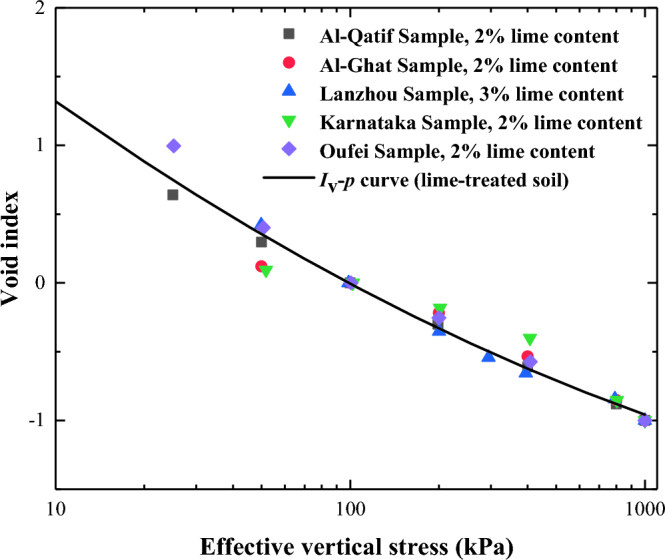
Comparison between verification results and *I*_v_-*p* curves of lime-treated soil.

Figure 17 shows a comparison between the *I*_v_-*p* curves of lime-treated soil and the validation results. The results indicate that the oedometer test outcomes for the five samples align well with the fitted curves. The *I*_v_-*p* curves of lime-treated soil derived in the article successfully predict the compression characteristics of the samples with lime content ranging from 0 to 3%.

## Conclusion

The findings of 60 groups of oedometer tests for a lime treated high-water-content slurry in Southeast China are presented in this paper. The compression curve of the slurry was measured in the pressure range of 1–800 kPa and its yield stress was analysed.

Using the log(1 + *e*) − log ${\sigma }_{\text{v}}^{\prime}$ compression relationship to analyse the soil’s compression curve it is evident that lime treatment promotes the formation of a stable soil structure in the soil and imparts the yield stress to the clay soils. The yield stress of the lime-treated soil increased with an increase in lime content and significantly decreased with an increase in water content. Further analysis of the yield stress revealed that the optimal lime content of the soil varied significantly with the water content.

Based on the regression equation of yield stress ${\upsigma }_{y}^{\prime}$ and normalised parameters ${e}_{0}/{e}_{L}$ proposed by Hong^19^, this paper presents an empirical formula suitable for lime-treated soil and an empirical parameter *L* reflecting the effect of lime treatment. The new formula combines the yield stress of lime-treated soil with the original remodelled soil parameters to obtain a good fit.

A normalised compression curve for lime-treated slurry with 1% to 3% lime content was established, and a simple method was proposed to predict the modified compression index of lime-treated slurry in pre-yield state. Based on this, the preliminary evaluation of compression characteristics of lime-treated slurry can be conducted without oedometer testing.

## Data Availability

The data that support the findings of this study are available on request from the corresponding author upon reasonable request.
